# New Aspects of Sarcomas of Uterine Corpus—A Brief Narrative Review

**DOI:** 10.3390/clinpract11040103

**Published:** 2021-11-22

**Authors:** Stoyan Kostov, Yavor Kornovski, Vesela Ivanova, Deyan Dzhenkov, Dimitar Metodiev, Rafał Watrowski, Yonka Ivanova, Stanislav Slavchev, Dimitar Mitev, Angel Yordanov

**Affiliations:** 1 Department of Gynecology, St. Anna University Hospital, Medical University of Varna, 9002 Varna, Bulgaria; drstoqn.kostov@gmail.com (S.K.); ykornovski@abv.bg (Y.K.); yonka.ivanova@abv.bg (Y.I.); st_slavchev@abv.bg (S.S.); 2 Department of General and Clinical Pathology, Faculty of Medicine, Medical University Sofia, 2 Zdrave Str., 1432 Sofia, Bulgaria; veselaivanovamd@gmail.com; 3 Department of General and Clinical Pathology, Forensic Medicine and Deontology, Division of General and Clinical Pathology, Faculty of Medicine, Medical University—Varna “Prof. Dr. Paraskev Stoyanov”, 9002 Varna, Bulgaria; dzhenkov@gmail.bg; 4 Clinical Pathology Laboratory, MHAT “Nadezda” Women’s Health Hospital, 1373 Sofia, Bulgaria; dr.dmetodiev@yahoo.com; 5 Neuropathological Laboratory, University Hospital “Saint Ivan Rilski”, 1431 Sofia, Bulgaria; 6 Faculty Associate, Medical Center—University of Freiburg, 79106 Freiburg, Germany; Rafal.Watrowski@gmx.at; 7 University Hospital SBALAG “Maichin Dom”, Medical University Sofia, 1000 Sofia, Bulgaria; dr.dimitur.mitev@gmail.com; 8 Department of Gynecologic Oncology, Medical University Pleven, 5800 Pleven, Bulgaria

**Keywords:** uterine leiomyosarcoma, low-grade endometrial stromal sarcoma, high-grade endometrial stromal sarcoma, undifferentiated uterine sarcoma, uterine adenosarcoma

## Abstract

Sarcomas of the uterine corpus are rare malignant neoplasms, which are further classified into mesenchymal tumors, and mixed (epithelial plus mesenchymal) tumors. The main issues concerning these neoplasms are the small number of clinical trials, insufficient data from evidence-based medicine, insignificant interest from the pharmaceutical industry, all of which close a vicious circle. The low frequency of these malignancies implies insufficient experience in the diagnosis, hence incomplete surgical and complex treatment. Additionally, the rarity of these sarcomas makes it very difficult to develop clinical practice guidelines. Preoperative diagnosis, neoadjuvant and adjuvant chemoradiation, target and hormone therapies still raise many controversies. Disagreements about the role and type of surgical treatment are also often observed in medical literature. There are still insufficient data about the role of pelvic lymph node dissection and fertility-sparing surgery. Pathologists’ experience is of paramount importance for an accurate diagnosis. Additionally, genetics examinations become part of diagnosis in some sarcomas of the uterine corpus. Some gene mutations observed in uterine sarcomas are associated with different outcomes. Therefore, a development of molecular classification of uterine sarcomas should be considered in the future. In this review, we focus on the epidemiology, pathogenesis, pathology, diagnosis and treatment of the following sarcomas of the uterine corpus: leiomyosarcoma, low- and high-grade endometrial stromal sarcomas, undifferentiated sarcoma and adenosarcoma. Uterine carcinosarcomas are excluded as they represent an epithelial tumor rather than a true sarcoma.

## 1. Introduction

Sarcomas of the uterine corpus are malignant neoplasms, which are further classified into mesenchymal tumors and mixed (epithelial plus mesenchymal) tumors [1,2]. The main issues concerning these neoplasms are the small number of clinical trials, insufficient data from evidence-based medicine, insignificant interest from the pharmaceutical industry, all of which close a vicious circle. The low frequency of these malignancies implies insufficient experience in the diagnosis, hence incomplete surgical and complex treatment. Some uterine sarcomas (leiomyosarcoma, high-grade endometrial stromal sarcoma, undifferentiated sarcoma, adenosarcoma with sarcomatous overgrowth) have poor prognosis, whereas others (low-grade stromal sarcoma, adenosarcoma without sarcomatous overgrowth) are associated with good prognosis [2]. Surgical treatment is the first-line approach, as there are still insufficient data about neoadjuvant treatment [1,2]. Moreover, the role of pelvic lymph node dissection, adjuvant chemoradiation, hormone and target therapies remain controversial [1,2]. Pathologists’ experience is of paramount importance for an accurate diagnosis. Additionally, genetics examinations become part of diagnosis in some sarcomas of the uterine corpus. In this review, we focus on the epidemiology, pathogenesis, pathology, diagnosis and treatment of the following sarcomas of the uterine corpus: leiomyosarcoma, low- and high-grade endometrial stromal sarcomas, undifferentiated sarcoma and adenosarcoma. Uterine carcinosarcomas are excluded as they represent an epithelial tumor rather than a true sarcoma [1].

## 2. Uterine Sarcomas

### 2.1. Uterine Leiomyosarcoma (ULMS)

#### 2.1.1. Epidemiology

ULMS is a malignant mesenchymal tumor of myometrial smooth-muscle derivation, which represents 60% of all uterine sarcomas and accounts for 1–2% of uterine malignancies. Its annual incidence rate is approximately 0.8 per 100,000 women. It is the most common type of uterine sarcomas originating from the myometrium [3,4,5,6]. It is more common among people of the African-American race, those that have been exposed to prolonged use of tamoxifen for over 5 years and women over 40 years of age, with incidence increasing rapidly after age 50 [5,7]. Moreover, there is an increased risk of ULMS for patients with p53 gene mutations, for those who have been exposed to radiotherapy for childhood cancers, and those with certain inherited genetic syndromes, including hereditary retinoblastoma and Li-Fraumeni syndrome [5,6,7].

#### 2.1.2. Clinical Presentations

The most common presenting symptoms for women with ULMS include enlarged uterus, abnormal vaginal bleeding, abdominal pain, vaginal discharge, urinary frequency, anaemia (without vaginal bleeding) and constipation [6,7,8]. An initial symptom could be abdominal pain due to hemoperitoneum caused by tumor rupture or extrauterine extension [8].

#### 2.1.3. Pathogenesis

Generally, ULMS arises from a solitary lesion, though multiple lesions are also documented [8]. There is no scientific evidence to provide an association between ULMS and leiomyoma. However, it is common to find both growths in a single specimen [7,8]. The exact pathophysiology of ULMS is not clearly understood and most studies continue to support extremely heterogeneous patterns [8]. It is supposed that chromosomal instabilities (complex numerical and structural chromosomal aberrations) are the hallmark of uterine smooth muscle tumors, but none are considered diagnostic [9]. The most frequently mutated gene is p53 (30%), followed by alpha thalassemia/mental retardation syndrome X-linked (ATRX) (25%), mediator complex subunit 12 (MED12) (20%), retinoblastoma protein (RB1) and breast cancer gene-2 (BRCA2) [3,5,10,11]. Vascular endothelial growth factor (VEGF) and platelet-derived growth factor (PDGF) are expressed in ULMS [5].

#### 2.1.4. Pathology

**Figure 1:**
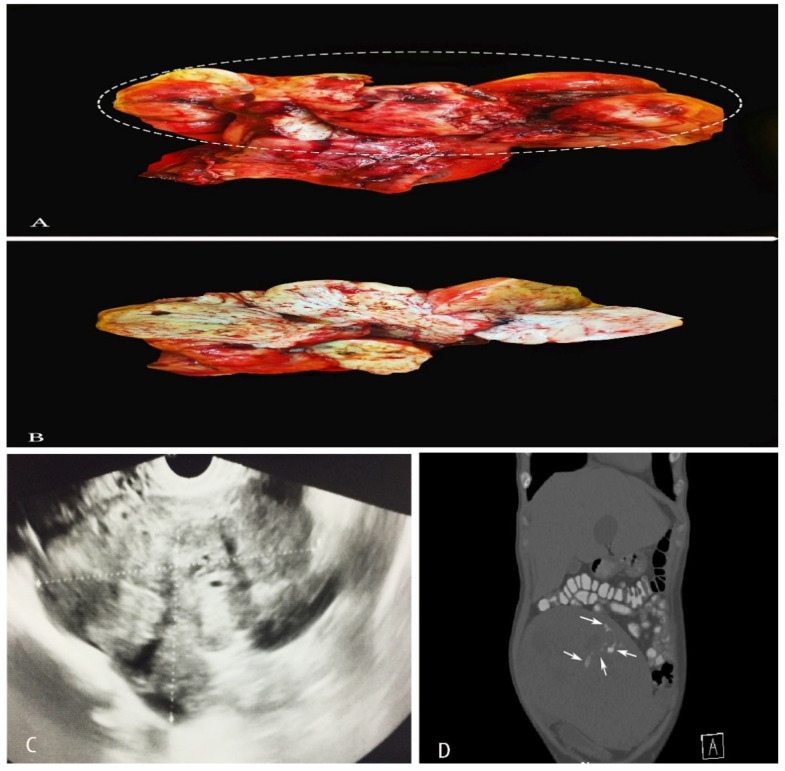
(**A**,**B**)—A case of uterine leiomyosarcoma with coexisting subserous and intramural leiomyomas. The ellipse shows the sarcomatous part of the specimen. (**C**)—Vaginal ultrasonography appearance of ULMS. (**D**)—CT appearance of ULMS. White arrows show areas of necrosis.

Grossly, ULMS often appears greyish yellow or pink, and with a soft or fleshy consistency (Figure 1) [4]. ULMS has poorly defined margins compared to its benign counterpart [5]. Haemorrhagic and necrotic areas are also often encountered [4]. The true tumor cell necrosis is characterized with an irregular angulated border, a sharp interface between viable and necrotic cells, the presence of apoptotic or nuclear debris at the interface and atypical ghost cells [4,5,6,7,8].

ULMS is not graded, as there is no correlation between high-grade histology and overall survival [6]. Differential diagnosis with benign variant leiomyomas (i.e., cellular leiomyoma, mitotically active leiomyoma, leiomyoma with bizarre nuclei, and leiomyoma with infarct-type necrosis) could confuse pathologists [6].

Microscopically, ULMS includes three subtypes: spindle cell/conventional, epithelioid, and myxoid [9].

**Figure 2:**
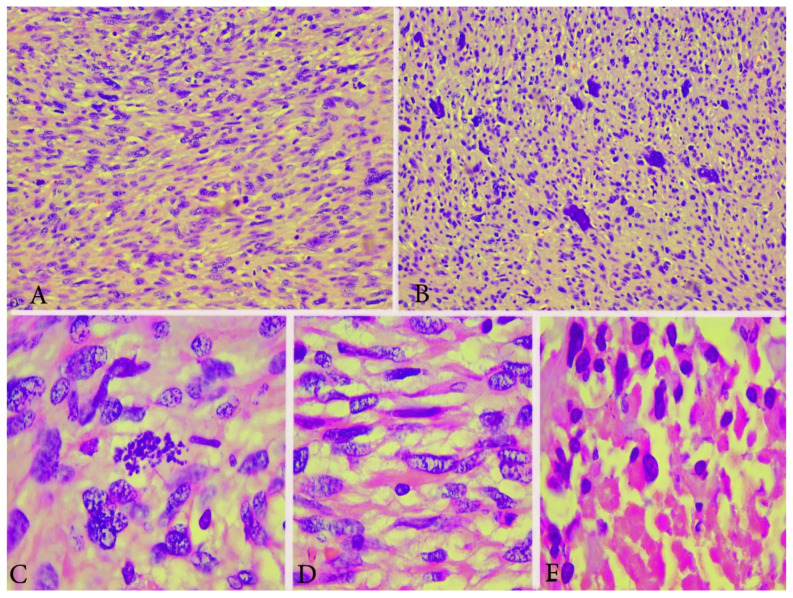
Microscopical features of spindle cell type ULMS (the same case from Figure 1). (**A**) Numerous atypical mitoses H&Ex200. (**B**) Area with plenty of pleomorphic cells H&Ex200. (**C**) Atypical mitoses H&Ex1000. (**D**) Atypical spindle cells H&Ex1000. (**E**) Area of tumor necrosis H&Ex1000.

Spindle cell-type/conventional ULMS—high mitotic index (>10 mitoses/10 high power fields and tumor cell necrosis), spindled/fascicular cells with moderate-to-severe pleomorphism, infiltrative border and the possible presence of multinucleated cells, myometrial invasion [9] are shown in Figure 2.

Epithelioid and myxoid ULMS are rare subtypes, which may have mild or focal nuclear pleomorphism and a lesser degree of mitotic activity than what is seen in the typical ULMS [6]. An abundant myxoid stroma is typical for myxoid ULMS, whereas round or polygonal cells with eosinophilic or clear cytoplasm are presented in epithelioid ULMS [9].

**Figure 3:**
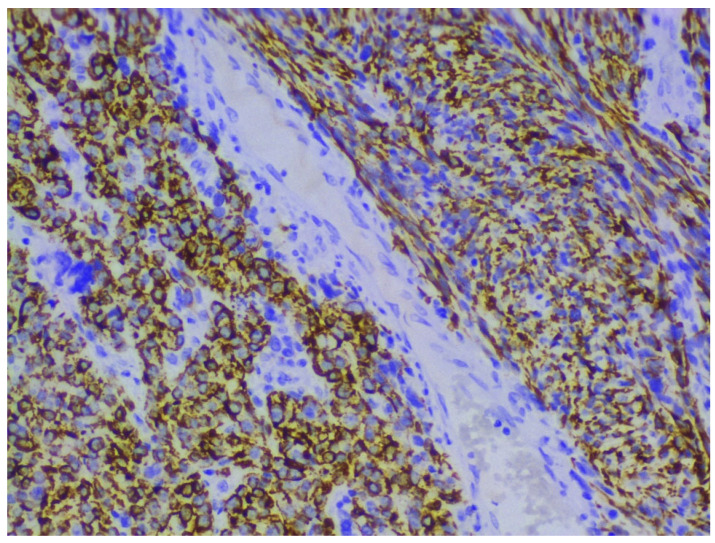
Immunohistochemical features of ULMS (the case from Figure 1 and Figure 2). Desmin (+) diffuse cytoplasmic expression in tumor cells H&Ex200.

Immunohistochemical staining with smooth muscle actin, desmin and h-caldesmon shows positive reaction in tumor cells, and p53, p16, Progesterone and Ki-67 help to differentiate ULMS from leiomyoma [5,12,13,14,15] (Figure 3). Estrogen and progesterone receptor expression is lower compared to leiomyoma [5,12,13,14,15]. 

#### 2.1.5. Diagnosis

There is mounting interest in the preoperative diagnosis of ULMS. Currently, no clinical symptom, laboratory test or imaging study can provide effective preoperative diagnostic modalities for ULMS [6,16]. Furthermore, differential diagnosis between ULMS and uterine leiomyoma is still a clinical challenge as all symptoms are often similar [5].

##### Imaging Modalities

On vaginal or abdominal sonography, large heterogeneous masses with areas with central vascularity or necrosis could be observed in cases of ULMS [5] (Figure 1). Computed tomography (CT) is inferior to magnetic resonance imaging (MRI), as it does not offer adequate contrast resolution for delineating focal myometrial masses. However, it could reveal areas of necrosis [5,17] (Figure 1). Positron emission tomography/computed tomography (PET/CT) is used for the detection of metastases and recurrence [17]. On magnetic resonance imaging (MRI), hemorrhage appears as increased signal intensity on both T1- and T2-weighted images, while decreased SI on T1-weighted images and increased SI on T2-weighted images show necrosis [17]. Contrast-enhanced MRI is superior to diffusion-weighted MRI in differentiating ULMS and leiomyomas [6]. Hata et al. observed that, on Color and pulsed Doppler findings, peak systolic velocity was significantly higher in uterine leiomyosarcoma compared to uterine myomas [18]. Authors concluded that the peak systolic velocity could be a useful marker for the preoperative differential diagnosis of uterine sarcoma [18].

In our opinion, there is no imaging modality which can differentiate ULMS from uterine leiomyoma, as both have similar findings on imaging. However, in cases with a newly diagnosed or enlarged pelvic mass, the patients should undergo a pelvic ultrasound by an experienced sonographer as an initial imaging study.

##### Laboratory Tests

Some studies have shown that levels of serum lactate dehydrogenase (LDH) total activity and its isoenzymes may be relevant in preoperative diagnosis of ULMS [19,20,21]. Nagai et al. presented a study of 63 patients, 15 diagnosed with uterine sarcoma and 48 with benign tumors. The authors concluded that LDH values, and MRI and endometrial cytology findings, are significant predictors of uterine sarcoma in both groups [19]. In this respect, Song et al. reported that the positivity rates for LDH-A and LDH-D were significantly higher in patients with uterine sarcoma compared with those with uterine myoma [20]. Nishigaya et al. concluded that a combination of LDH, D-dimer and C-reactive protein might be useful for distinguishing ULMS from especially degenerated or atypical leiomyoma [21]. 

In conclusion, LDH total activity and its isoenzymes may play a role in the preoperative evaluation of suspect uterine mass. However, further studies are necessary to determine its actual reliability.

##### Endometrial Samplings

Endometrial biopsy or curettage may detect uterine ULMS in a substantial proportion of cases—approximately in 25% to 50% of cases [7,8]. Therefore, a negative biopsy does not preclude the diagnosis until the complete hysterectomy specimen is examined [6]. The detection of ULMS by endometrial sampling is higher in cases of endometrial involvement by the tumor [7,8].

#### 2.1.6. Prognostic Factors

The majority of prognostic factors of ULMS are summarized in Table 1 [5,6,7,8,22]. In one of the biggest studies, Hosh et al. analysed epidemiology, prognostic factors and surveillance of 13,089 patients with uterine sarcomas. Authors concluded that older age, Afro-American race, advanced tumor grade and stage were associated with worsened survival [22]. Special attention should be paid about morcellation of ULMS. Power morcellation of unexpected ULMS during laparoscopic myomectomy or hysterectomy is a poor prognostic sign. It is associated with a high recurrence rate and distant metastases. Recently, in one of the largest studies, Nobre et al. compared 107 patients with ULMS who underwent total hysterectomy (non-morcellated) versus 45 patients with ULMS who underwent total hysterectomy with morcellation. Median progression-free survival and median overall survival were inferior in the morcellated group. Moreover, patients who underwent morcellation had nearly a 4-fold increase in peritoneal recurrence vs. the non-morcellated group [23]. Currently, the U.S. Food and Drug Administration (FDA) recommended performing laparoscopic power morcellation for myomectomy or hysterectomy only with a tissue containment system. Preoperative patient’s evaluation (different imaging modalities, cervical cancer screening, and endometrial tissue sampling) before considering morcellation should be performed. Furthermore, The FDA stated that morcellation should be avoided in postmenopausal patients, older than 50 years, or candidates for removal of tissue (en bloc) through the vagina or via a mini-laparotomy incision [24,25].

FIGO staging of uterine ULMS and endometrial stromal sarcomas is shown in Table 2.

#### 2.1.7. Treatment

##### Surgical Treatment

In cases of ULMS confined to the uterus, total abdominal hysterectomy (TAH) without lymph node dissection (LND) is recommended. The procedure could be performed by open surgery or minimally invasive techniques. The incidence of lymph node metastases is approximately 5% to 11% and lymphadenectomy does not impact overall survival (OS). Moreover, hematogenous metastases are typically presented in patients with ULMS—the most common site of initial metastasis is the lung, followed by the peritoneal cavity, liver parenchyma, brain, and bone [4,5,6,7,8,9]. However, if bulky notes are presented, LND should be performed as a part of optimal cytoreduction. Ovarian preservation (OP) is not associated with worse OS or a high recurrence rate. It may be considered for pre-menopause women with early-stage disease (I–II). Nasioudis et al. analysed 800 women with ULMS, of which 29.6% underwent TAH with OP. Authors concluded that women with stage I ULMS and OP had better 5-year OS and cancer-specific survival compared to women, who underwent oophorectomy [27]. If ULMS is encountered on final pathology after myomectomy or supracervical hysterectomy, a completion procedure with TAH, trachelectomy, and/or bilateral salpingo-oophorectomy is recommended. Fertility-sparing procedures are not recommended [4,5,6,7,8]. For patients with unresectable ULMS, TAH should be performed only in select cases of palliation for uncontrollable uterine bleeding when other conservative management approaches have failed [7].

##### Adjuvant Radiation Therapy (ART)

ART does not improve OS but may improve local control. It is recommended in cases of locally advanced tumors confined to the pelvis. A combination of ART and adjuvant chemotherapy impact disease-free survival for 3 years, but do not affect overall 3–5-year survival rate [5,6,7,8]. Generally, ART is not recommended. A randomized phase III clinical trial showed that ART did not improve OS of patients with stage I and II ULMS [28]. ESMO guidelines concluded that radiation therapy may be discussed with patients in cases with higher stages considering special risk factors, such as mitotic count, age and necrosis of the tumor [29].

##### Adjuvant Chemotherapy (ACT)

There is no proven benefit of ACT [6]. There are limited data and many controversies about the role of ACT in early-stage ULMS. The majority of studies stated that ACT did not influence OS and disease-free survival (DFS), and it is not a significant predictor for disease-specific survival or recurrence. In Stage I ULMS, observation, systemic therapy or estrogen blockade in case of ER-positive tumors are recommended [4,5,6,7,8,30]. Currently, randomized phase III clinical trial compared adjuvant gemcitabine-docetaxel for 4 cycles followed by 4 cycles of doxorubicin with observation for patients with stage I ULMS. The clinical trial was not completed due to lack of accrual. However, authors observed that OS and recurrence-free survival data do not show superior outcomes with adjuvant chemotherapy [30,31]. A phase III randomized clinical trial compared adjuvant chemotherapy with doxorubicin, ifosfamide and cisplatin followed by RT with RT alone in completely resected uterine sarcomas. The 3-year DFS was 55% for adjuvant chemotherapy vs. 41% for RT alone [30,32]. 

The first-line treatment is monotherapy with doxorubicin as it is well tolerated and less toxic than other chemotherapy agents. Recommended combination regimens include doxorubicin/ifosfamide and doxorubicin/dacarbazine. Ifosfamide monotherapy treatment is under investigation. Gemcitabine treatment alone or a combination of gemcitabine/dacarbazine and gemcitabine/vinorelbine are other recommended regimes [4,5,6,7,8]. 

In advanced stage of the disease, target therapies could be initiated (Pazopanib, Temozolomide, Trabectedin, Eribulin). Trabectedin treatment may improve the quality of life, and it leads to long-term stabilization of the tumor. Pazopanib significantly increased median progression-free survival (PFS), but not OS. Bevacizumab, a monoclonal antibody against VEGF, does not improve OS and PFS [4,5,6,7,8]. Immunotherapy with programmed death-ligand 1 (PD-L1) inhibition in ULMS showed no benefits [30].

##### Metastatic and Recurrent ULMS

If it is feasible, secondary cytoreduction is the first option in cases of metastatic sarcoma. Better outcomes may be expected in cases of prolonged time to recurrence (>12 months) and an isolated site of recurrence [4,5,6,7,8]. Burt et al. analysed 82 patients, who underwent pulmonary metastasectomy for metastases from sarcoma. The majority of patients had leiomyosarcoma. Authors found that patients with leiomyosarcoma and pulmonary metastasectomy had improved overall survival compared with those with nonleiomyosarcoma metastases [33]. Another study reported 65% 5-year survival in 45 patients undergoing pulmonary metastasectomy for metastatic uterine sarcoma. The majority of patients (38) had ULMS [34].

Doxorubicin is a preferable single agent for the treatment of metastatic ULMS, as it is less toxic than combination regimes [35]. Gemcitabine has had response rates as high as 21%, when it is used as a single agent. Other recommended single agents are dacarbazine, epirubicin, gemcitabine, pazopanib, and temozolomide [35]. Combination chemotherapy had better response rates compared to single agents for patients who can tolerate such an aggressive regime [35]. In 2002, Hensley et al. published a phase II study examining the efficacy of gemcitabine plus docetaxel for the treatment of unresectable ULMS [36]. In 2008, Hensley et al. published a phase II study, examining the role of gemcitabine plus docetaxel as a first-line therapy in metastatic uterine leiomyosarcoma [37]. Authors concluded that the combination of gemcitabine–docetaxel should be a standard first-line treatment for patients with unresectable and metastatic ULMS [35,36,37]. In another phase III study, authors reported that the addition of bevacizumab to gemcitabine–docetaxel for first-line treatment of metastatic ULMS did not improve PFS and OS [38].

A retrospective multicenter study of the Spanish ovarian cancer research group concluded that trabectedin could be used as a second- or third-line treatment such as ULMS. Authors stated that trabectedin showed clinical benefit in patients with recurrent/metastatic ULMS and it could be given after failure to an anthracycline-based regimen [39]. In 2017, Hensley et al. published a phase III randomized clinical trial, analysing the efficacy and safety of trabectedin or dacarbazine in patients with advanced ULMS after failure of anthracycline-based chemotherapy. Authors observed that, after prior anthracycline therapy, trabectedin treatment resulted in significantly longer PFS compared to dacarbazine. However, there were no differences in OS [40].

Pelvic radiation could be utilised in cases of unresectable loco-regional relapse. Radiation therapy may be considered after pelvic metastasectomy for better local control, but there will be no benefit for OS.

**Figure 4:**
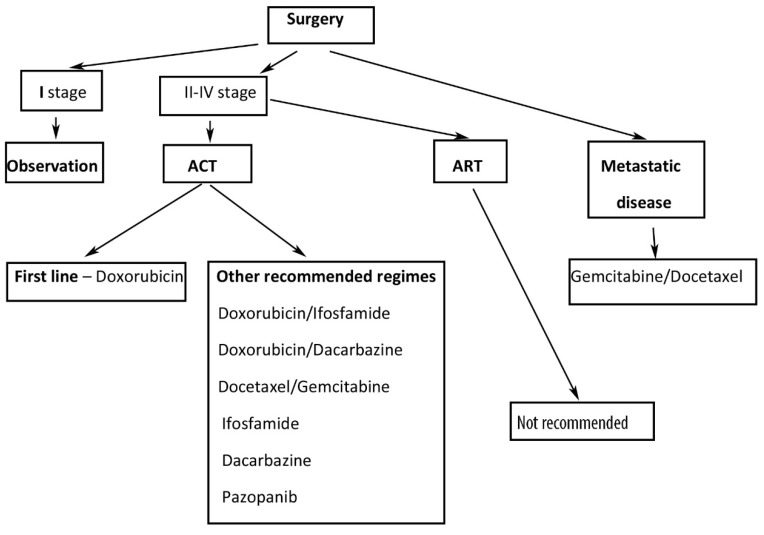
Suggested treatment of ULMS. ACT—adjuvant chemotherapy, ART—adjuvant radiation therapy.

Suggested treatment of ULMS is shown in Figure 4.

### 2.2. Endometrial Stromal Sarcomas (ESSs)

Endometrial stromal tumors are of endometrial stromal origin and account for less than 2% of uterine tumors. In accordance with the current World Health Organization (WHO) classification 2020, endometrial stromal tumors include endometrial stromal nodule, low-grade endometrial stromal sarcoma (LGESS), high-grade endometrial stromal sarcoma (HGESS) and undifferentiated uterine sarcoma (UUS) [3,41]. Endometrial stromal nodule is a benign tumor and it is not the topic of the present article. LGESS and HGESS are rare among uterine malignancies, together accounting for 1% of uterine malignancies and 10% of uterine sarcomas [42]. As mentioned above, the FIGO staging of ESSSs is the same as leiomyosarcoma FIGO staging.

#### 2.2.1. LGESS

LGESS, also previously known as endolymphatic stromal myosis, is the second most common type of uterine sarcomas (second to ULMS) [43,44]. Although LGESS affects women primarily in the perimenopausal age group, cases of its occurrence among young women and adolescents have been described [41,44,45,46,47]. Patients with obesity, tamoxifen use, diabetes mellitus, those administered estrogen and a history of radiotherapy are at risk of developing LGESS [41]. The majority of patients presented with pelvic pain, abnormal uterine bleeding and dysmenorrhea. In the beginning, the disease could be asymptomatic as nearly one-third of patients present with symptoms related to extrauterine spread [44]. Approximately 50% of extrauterine spread cases of LGESS are associated with endometriosis [44,46,47]. The common extrauterine sites of involvement associated with the existence of endometriosis are the abdominopelvic sites, specifically the peritoneal surfaces, bowel wall, ovaries, pelvis, vagina, urinary bladder, retroperitoneum, lymph nodes, and fallopian tubes [48]. Some authors even define LGESS as “endometrioid” stromal sarcomas and exclude the uterus as the primary site [47,49]. Diagnosis and staging are the same as for ULMS. However, the probability to establish the diagnosis of LGESS preoperatively by endometrial sampling is higher than that of ULMS [44,45,46,47,48,49,50,51,52]. Moreover, in cases where LGESS arises as endometrial polyp, the diagnosis is often achieved by diagnostic curettage [44]. The 5-year survival rate is over 90% for Stage I–II and around 50% for Stage III–IV [44]. Unfortunately, it exhibits slow clinical progression with repeated local recurrences [43]. The recurrence risk of LGESS is high (50%), even in Stage I—median time to recurrence 65 months [46,47].

##### Pathogenesis and Pathology

Different chromosomal aberrations have been detected in LGESS of which JAZF1-SUZ12 genes fusion is the most common, followed by JAZF1-PHF1, EPC1-PHF1 and MEAF6-PHF1 [3,53].

Grossly, the LGESS are soft tan to yellow, smooth-surfaced polyps or nodules (intramural or submucosal) that could be partly infarcted and hemorrhagic (tumor size ranges from 1 cm to 25 cm, with a mean of 8–11 cm). Although it is usually with ill-defined borders and worm-like permeation within the myometrium and blood vessels, some tumors might appear relatively circumscribed [41,44,47,48]. Areas of hemorrhages, necrosis and cystic degeneration are frequently observed [41].

Typical histological features of LGESS are the “tongue-like” patterns of myometrial and lymphovascular invasion [44]. Cytomorphologically, the LGESS consists of small cells with round to oval uniform nuclei, scant cytoplasm and low mitotic activity (<5/10 high power fields), and without necrosis. Higher mitotic activity (10–15 mitotic figures per 10 HPF in most active areas) does not exclude the diagnosis [43,44,47]. 

This may exhibit sex cord-like, epithelioid and glandular differentiation. Other features helpful in the diagnosis include an arborizing vascular pattern, foam cells with necrosis and rope-like collagen [41,42,43,44,45,46,47].

**Figure 5:**
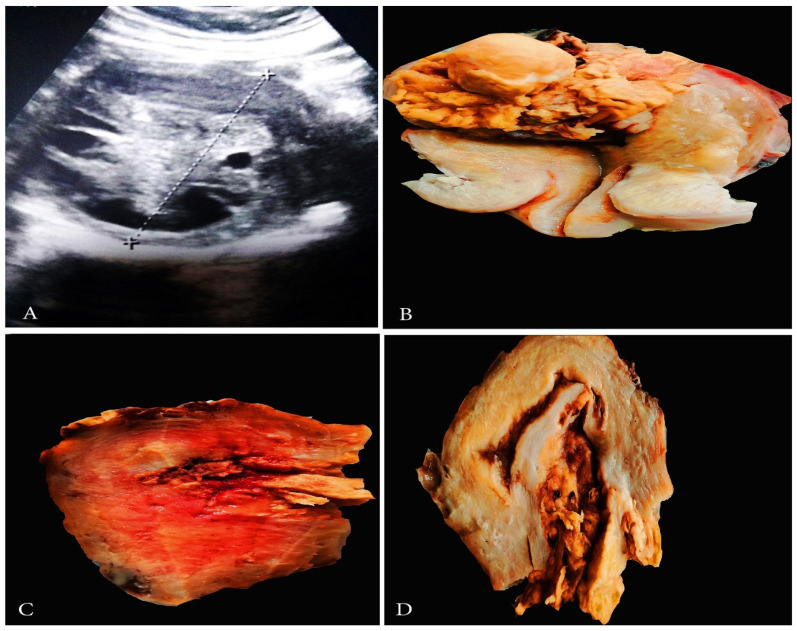
Ultrasonographic and grossly features of LGESS. (**A**) Abdominal ultrasonography of LGESS; (**B**–**D**) Gross appearance of LGESS.

**Figure 6:**
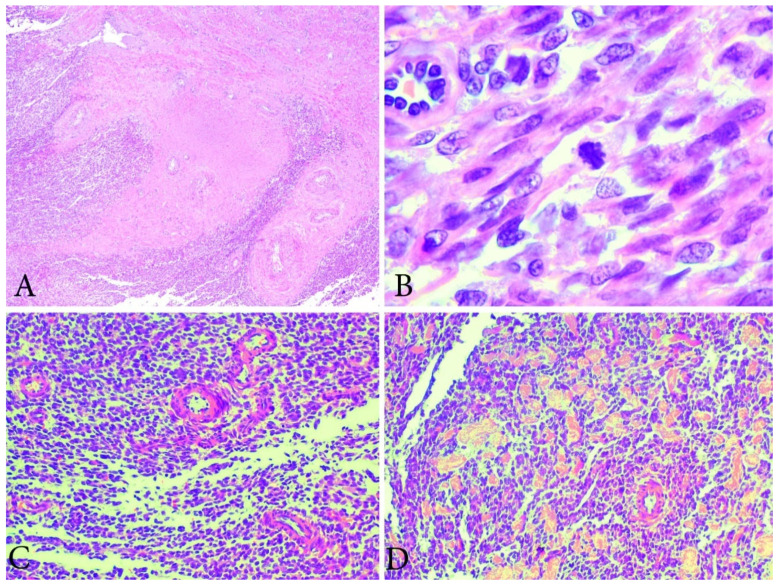
Microscopical features of LGESS (the same case from Figure 5). (**A**) Tongue-like outgrowths of myometrial invasion. H&Ex100. (**B**) Low mitotic activity in LGESS. H&EX400. (**C**) The cells resemble proliferative-phase endometrial stroma, being bland with interspersed small arterioles. H&Ex200. (**D**) Focal area with hyalinization. H&Ex200.

It is important to notice that distinguishing LGESS and endometrial stromal nodule on curettage specimen is not possible [41]. Immunohistochemically, the LGESS tumor cells are strongly positive for CD10. Actin and h-Caldesmon are positive in areas with smooth muscle differentiation, whereas inhibin, calretinin, Melan-A, and CD99 are usually positive in rare areas with sex cord-like differentiation [44,48]. In 67% of cases, strong nuclear immunoreactivity with beta-catenin is observed [43]. In the majority of cases, these sarcomas are positive for estrogen and progesterone receptors [43,44,48] (Figure 5 and Figure 6).

##### Prognostic Factors

Unfavorable prognostic factors include: advanced stage, increased age of the patient, non-surgical treatment, morcellation [41,42,43,44,45,46]. There is no consensus regarding lymph node involvement. Chan et al. detected in 9.9% pelvic lymph node metastases of 282 women who had lymphadenectomy. The authors concluded that lymph node involvement is associated with poorer survival rates [53]. Other authors concluded that the LND might reduce the number of recurrences in the pelvis. However, pelvic and paraaortic LND do not appear to have any influence on OS [46,52,54]. The relevance of mitotic activity count and overexpression of p-53 as prognostic markers is unclear [46].

##### Treatment

TAHBSO is the standardized treatment modality in the treatment of patients with LGESS [44]. Optimal cytoreduction is performed for patients with extra-uterine spread [50]. Preserving the ovaries in premenopausal women with LGESS is controversial, but most studies concluded that it is associated with a high recurrence rate and decrease OS. Fertility-sparing surgeries (polypectomy and adjuvant hormone therapy) could be considered as an option in young patients with Stage I LGESS [46,55]. ART improves locoregional control but does not affect OS and PFS [42]. NCCN recommended observation after TAHBSO for patients with Stage I LGESS. No additional adjuvant therapy is needed [52]. NCCN also stated that, for Stage II-IV TAHBSO, anti-estrogen hormone therapy and external beam ART could be performed [56]. ACT is not recommended as it does not increase OS and PFS [46,47], as LGESS overexpress both estrogen and progesterone receptors studies as shown in benefits of hormone treatment. Adjuvant hormone therapy includes progestin-based hormones (megestrol acetate, medroxyprogesterone), gonadotropinreleasing hormone analogs. Treatment with aromatase inhibitors is also recommended, but it is not used in cases of estrogen negative LGESS or in pre-menopausal women [46,50,57]. Hormonal treatment is mainly administered for patients with advanced-stage (III or IV) or residual tumors [50]. In conclusion, progestin-based hormones and aromatase inhibitors have a therapeutic role in LGESS treatment, whereas estrogen-based hormone replacement therapy is contraindicated [57]. Target therapy treatment (PDGFR, EGFR, VEGFR, and HDAC inhibitors) has been used sporadically and is under consideration [47].

#### 2.2.2. HGESS

In 2014, the WHO officially reintroduced HGESS based on the identification of YWHAE-NUTM2A/B gene fusion (also known as YWHAE-FAM22A/B). The fusion of these two genes causes activation of 14-3-3 oncoprotein as a recurrent event in this more malignant subgroup of tumors [44,58,59]. Therefore, patients with HGESS experience earlier and more frequent recurrences (often <1 year) [57]. The PFS (7–11 months) and OS (11–23 months) of HGESS are worse than those for LGESS [58,59]. YWHAE rearrangements have not been observed in other gynecological malignancies [47]. Other gene fusions of patients with HGESS are BCOR ITD, ZC3H7B-BCOR and EPC1-BCOR [41,56]. The real incidence of HGESS is not known, as in the past at least some of these tumors may have been confused with undifferentiated uterine sarcoma [41]. Epidemiology, symptoms, staging and diagnosis do not differ from LGESS. Tumor marker levels of CA-125 may correlate with the progression of the disease [59]. HGESS has a poor prognosis, as more than 50% of patients will present with an advanced stage of the disease. Lung metastases are most common [41,44,58,59,60,61,62].

##### Pathology

Grossly, HGESS is a pink-to-yellow-tan polypoid friable tumor with extensive invasion through the myometrium [60]. Cases of HGESS appearing as myoma nascens or multiple intrauterine tumors have been described [47,60,61].

**Figure 7:**
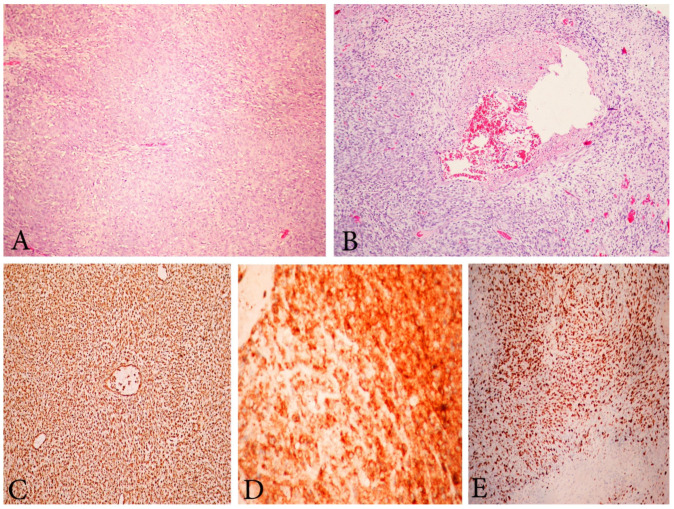
Microscopic feature of HGESS. (**A**) High-grade endometrial stromal sarcoma. H&Ex200. (**B**) High-grade endometrial stromal sarcoma with vascular thrombosis. H&Ex200. (**C**) High-grade endometrial stromal sarcoma, immunohistochemical positivity for Vimentin ×200. (**D**) Positive immunohistochemical reaction for CD10 ×400. (**E**) High Ki-67 proliferative index ×200.

Histological features include tongue-like permeation, high mitotic activity (>20–30 mitoses/10 HP fields), variable micronecrosis, lymphovascular invasion, mild-to-severe nuclear pleomorphism (Figure 7) [60,61]. Microscopically, in patients with gene fusion ZC3H7B-BCOR, focal cytoplasmic signet ring-like cell change, myxoid stromal change and osseous metaplasia could be observed [60]. Immunohistochemically, HGESS with gene fusion ZC3H7B-BCOR are typically positive for cyclin D1 and diffuse positive for CD10 with variable estrogen and progesterone positivity, whereas BCOR ITD tumors are less positive for CD10 and diffuse positive for cyclin D, BCOR and negative for estrogen and progesterone. YWHAE-FAM22A/B fusion subtype tumors are negative for CD10 and positive for cyclin D1, BCOR, KIT, CD56 [3,63,64,65].

##### Prognostic Factors

The single most significant prognostic factor is tumor stage. Other prognostic factors are morcellation, age, surgery, chemotherapy and radiotherapy [42,66]. Unlike LGESS, lymph node involvement is an important contributor to the survival of HGESS. Lymph node metastases occurs in 22% of patients with HGESS [42,66]. However, LND does not affect PFS and OS [42,66].

##### Treatment

Currently, due to the rarity of the disease, there are many controversies about preferable treatment options. Therefore, treatment options should be stated as suggestions. TAHBSO is the standard surgical procedure. Optimal cytoreduction in the advanced stage is recommended [47]. A study conducted by Seagle et al. showed survival benefits of ACT and ART for HGESS [42]. Zhang et al. concluded that the combination of surgery with radiotherapy and chemotherapy might only improve the PFS of patients with early-stage disease [59]. Gemcitabine combined with docetaxel and doxorubicin or ifosfamide and doxorubicin monotherapy are the most widely used chemotherapy treatment options [56,60,62]. NCCN recommended observation for patient diagnosed with Stage I and TAHBSO followed by systematic therapy and/or external beam radiation therapy for advanced stages. Doxorubicin monotherapy is recommended as first-line treatment [56]. Target therapies are under consideration and only few case studies reported the effectiveness of Pazopanib (multitarget tyrosine kinase inhibitor) and Apatinib (inhibitor on the vascular endothelial cell membrane) [62,67]. Fertility-sparing surgery and adnexal preservation should not be considered, though conclusive data are not yet available [44,56]. Hormone therapy did not improve the prognosis of HG-ESS patients given the lack of hormone receptor expression in HGESS [59].

#### 2.2.3. Undifferentiated Uterine Sarcoma (UUS)

**Figure 8:**
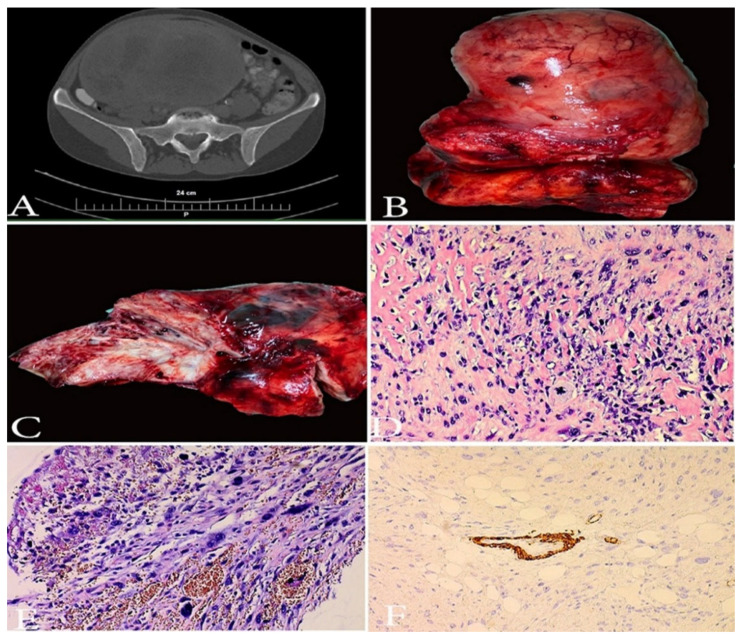
A case of undifferentiated uterine sarcoma. (**A**)—CT image of UUS. (**B**,**C**)—Gross appearance of UUS—presence of necrosis. (**D**,**E**)—spindle and pleomorphic cells, brisk atypical mitotic figures, infiltration in periuterine fat tissue, H&Ex200. (**F**)—immunohistochemistry: negative reaction in tumor cells for h-caldesmon, positive internal control in the muscular layer of the blood vessel, ×200. Other Immunohistochemical staining, which were performed in the selected case—(smooth muscle actin (-), ER/PR (-), WT1 (-), PAX 8 (-), CD-10 weakly positive, p53 (+). Elevated LDH, D-dimer and C-reactive protein serum levels were observed.

**Figure 9:**
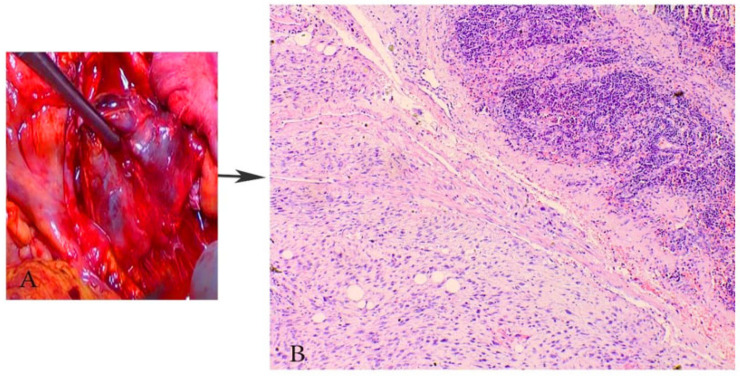
Paraaortic lymph node metastases in a patient with UUS (the same case from Figure 8). (**A**) macroscopic appearance of paraaortic lymph node metastases from UUS, (**B**) paraaortic lymph node metastases from UUS, H&Ex200.

UUS is an extremely rare and aggressive tumor with no specific line of differentiation that constitutes a diagnosis of exclusion [58,68,69,70]. The diagnosis should be performed after exclusion of other more common high-grade neoplasms (ULMS, carcinosarcoma, rhabdomyosarcoma, HGESS, LGESS, undifferentiated endometrial carcinoma and diffuse large B-cell lymphoma) (Figure 8). Approximately 60% of patients presented at advanced stage of the disease have a dismal prognosis (<2-year survival) [58]. UUS may arise in the endometrium or myometrium and is composed of pleomorphic or uniform cells with a high mitotic index that show no evidence of stromal or smooth muscle derivation [68,69,70]. Tumor necrosis, lymphovascular space involvement and destructive myometrial invasion are also presented [68,69,70]. Studies reported that 70% of UUS were in fact misdiagnosed HGESS [70]. Mitotic index appears to have prognostic significance in UUS, as a mitotic index higher than 25 mitoses per 10 high-power fields (HPFs) has a negative prognostic impact on survival [58,68,69,70]. Loss of SMARCA4 expression (mutation is often associated with remodeling ATPase in cancer) has recently been detected in a subset of UUS [70]. SMARCA4-deficient UUS are clinically aggressive with a median age of occurrence—48 years [70]. However, in some cases of low mitotic count, estrogen and progesterone expression long-term survival could be achieved [71,72]. Immunohistochemically, UUS is positive for p53, p16 and variably positive for CD10; estrogen and progesterone receptors are weakly positive or negative; keratin is negative [58,71]. Immunohistochemistry for ZC3H7B-BCOR, YWHAE-NUTM2 (FAM22) and BCOR ITD is essential in order to exclude HGESS [3]. Similar to other endometrial stromal sarcomas, the treatment of UUS includes TAHBSO. Pelvic lymph node involvement occurs in approximately 33% of patients (Figure 9). However, LND should be considered only as part of a cytoreductive procedure, as it does not seem to impact OS [68]. The benefits of ACT and ART are not clear, though studies reported increases in OS after ART and improved disease-free survival after initiation of adjuvant therapy in early stages [68,73]. NCCN recommended the same treatment as HGESS [56]. There is a need for prospective studies evaluating cytostatic activity of doxorubicin in advanced UUS, as well as the application of targeted therapy [70].

**Figure 10:**
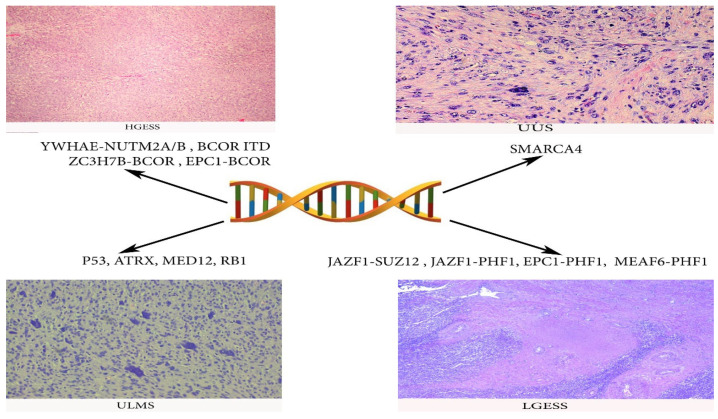
Gene mutations frequently observed in ULMS, LGESS, HGESS and UUS.

Genes mutations of ULMS, LGESS, HGESS and UUS are summarized in Figure 10.

## 3. Mixed Epithelial–Mesenchymal Uterine Tumors

### 3.1. Uterine Adenosarcoma (UAS)

#### 3.1.1. Definition, Epidemiology and Pathogenesis

UAS is a rare biphasic tumor composed of benign glandular epithelium and malignant stromal elements [74,75]. UAS accounts for <0.5 of all uterine malignancies and 5% of uterine sarcomas [75]. The incidence peak is in the 50 s and 60 s of the affected patients, though it could occur among young patients and adolescents [76]. It could be encountered in the uterine cervix. Patients with cervical manifestation of the tumor tend to be younger than the ones with UAS [76]. Although rare, these tumors could be identified in the ovary or in extra-uterine tissues [77]. It is stated that approximately 1–10% of adenosarcomas are thought to arise from the malignant transformation of endometriosis [74]. Cases of adenosarcomas arising due to endometriosis have been reported in medical literature—from adenomyosis of the uterus, bladder adenosarcoma arising from endometriosis, and adenosarcoma arising in an abdominal scar in a patient with a history of endometriosis [78,79,80]. Unlike uterine carcinosarcoma, UAS is a mesenchymal neoplasms where clonal genetic alterations are found in the sarcomatous but not in the epithelial components. UAS derives from neoplastic transformation of a Mullerian mesenchymal cell, which thus stimulates reactive growth of benign companion gland [3,81]. Most of the patients present with Stage I disease, with a 5-year OS of 60 to 80% [82]. Risk factors include endometriosis, tamoxifen use, pelvic radiation and prolonged estrogen exposure [77].

#### 3.1.2. Clinical Characteristics

Clinical features include abnormal vaginal discharge or vaginal bleeding, sometimes accompanied by an enlarged uterus. At advanced stages, women have abdominal pain and distension [74,75,76].

#### 3.1.3. Pathology

Grossly, UAS typically presents as a polypoid mass ranging from 1 to 20 cm. Cases of multiple polyps have been described [77]. The tumor can fill the entire uterine cavity and project into the endocervical canal [77,83]. The cut surface is solid, white to tan in color. Small cysts containing water and or mucoid fluid and papillary or polypoid projections into cystic spaces can often be appreciated macroscopically [82,83]. 

Currently, Müllerian adenosarcomas are not graded histologically as 50% of patients with the previously described low-grade UAS will develop disease recurrence. Therefore, UAS should not be considered as a tumor of low malignant potential [77,83]. 

**Figure 11:**
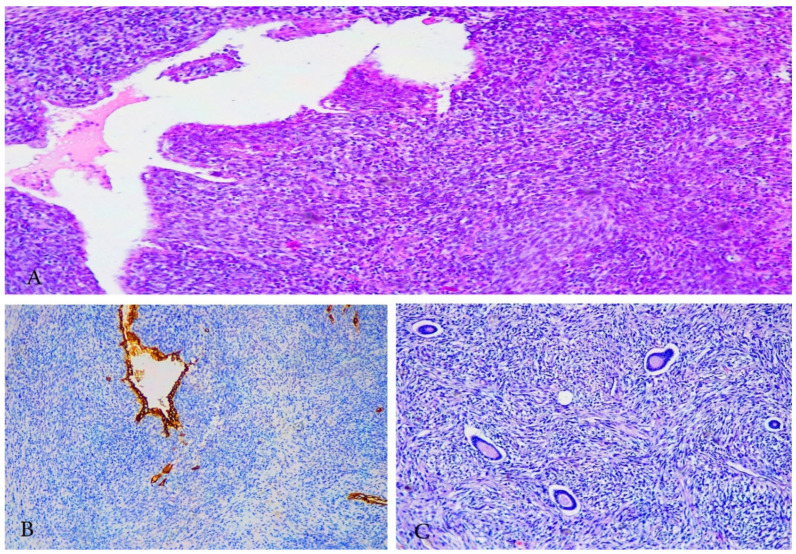
Microscopical features of adenosarcoma. (**A**,**C**) Benign epithelial component (endometrioid) and malignant mesenchymal component (stromal) around the glandular components; (**A**) H&Ex100, (**C**) H&Ex200. (**B**) Cytokeratin (+) in a benign epithelial component.

Low-power microscopic examination shows a biphasic tumor with admixed glands and prominent stroma throughout (Figure 11). Morphologic features include [75,82,83]:

- Broad leaf-like appearance, formed by the malignant stroma compressing the benign epithelium.
- The stroma is typically cellular, atypical, with periglandular stromal cuffing, a feature that distinguishes these tumors from endometrial or adenomatous polyps.
- Spindled/round cells are located around the glandular components and form peri-glandular cuffs.
- Mitotic activity ≥2 mitoses/10 HPFs (most noticeable in areas of peri-glandular cuffing).

Although mucinous, serous, and squamous epithelium components have been described, the epithelial component is usually endometrioid and metaplasia (squamous and mucinous are most common) is often observed [77,82]. Homologous and heterologous (most commonly rhabdomyosarcoma) sarcomatous components could be presented in the specimen [77,82,83]. If the sarcomatous component comprises more than 25% of the tumor, the term is called “Sarcomatous overgrowth”. It is a significant prognostic factor, as it is associated with deeper myometrial and lymphovascular invasion. Sarcomatous overgrowth has been described in 8–54% of cases [84].

There are no immunohistochemical markers pathognomonic for adenosarcoma [77]. The most common ones used are—CD10, ER, PR, AE1/AE3, vimentin, actin, WT1. Adenosarcoma with sarcomatous overgrowth shows loss of CD10 and PR [82,83].

#### 3.1.4. Diagnosis

Preoperative radiological and pathological findings are not specific enough to provide accurate diagnosis and seem to be difficult for radiologists and pathologists [75,77]. CT may show a well-demarcated mass that is hypointense/heterogeneous on T1 and multi-septated cystic appearance on T2 [77]. Tate et al. reported a retrospective study of 110 patients with UAS. The authors stated that 63% of initial imaging diagnoses and 53% of initial histologic diagnoses could indicate malignant neoplasms. Moreover, the preoperative pathological diagnosis of adenosarcoma was made in only 19% of endometrial biopsies and 41% of tumor biopsies [75].

#### 3.1.5. Prognostic Factors

Prognostic factors are summarized in Table 3 [74,77,78,79,80,81,82,83,84].

The most significant prognostic factors are: age, sarcomatous overgrowth, myometrial invasion, lymphovascular invasion, and lymph node involvement. Tumor necrosis and cellular atypia (asterisk in the table) could not be independent risk factors, as they are associated with sarcomatous overgrowth, high mitotic rate, myometrial invasion or necrosis [74,77,78,79,80,81,82,83,84]. Resection status and rhabdomyosarcoma components are possible prognostic factors [74,77,78,79,80,81,82,83,84]. 

FIGO staging of UAS is shown in Table 4 [76,77].

#### 3.1.6. Treatment

TAHBSO is recommended for the majority of patients with UAS [74]. Adnexes could be preserved in premenopausal patients, as adnexectomy is not associated with a higher survival benefit [74]. Deep myometrial invasion, large tumor, and sarcomatous overgrowth increase the presence of lymph node metastases [76]. There are no data showing that LND is associated with increased OS [74,76]. However, in cases of bulky lymph nodes, LND should be performed as a part of optimal cytoreduction [76]. There are controversies about fertility-sparing surgery (FSS). It is stated that FSS (polypectomy) is possible in the early stages (FIGO Stage I) and without a significant risk factor (sarcomatous overgrowth). We believe that FFS should be performed only in patients with Stage IA and without sarcomatous overgrowth, as myometrial invasion is associated with a high recurrence rate. The risks and benefits of the alternatives to the treatment should be discussed with patients and a strict oncological follow-up is necessary [85,86]. Similar to the other uterine sarcomas, there are no standardized ACT, ART or hormonal therapy regimens. However, it has been stated that patients at low risk of disease recurrence required observation alone, whereas for high-risk patients ACT may be recommended [76,77]. Some chemotherapeutic drugs—doxorubicin, ifosfamide, or gemcitabine/docetaxel, trabectedin—could be administered for patients with UAS and sarcomatous overgrowth [82].

#### 3.1.7. Recurrent or Metastatic Disease Treatment

The first treatment option is surgical resection of local recurrence and distant metastases. They occur in approximately 22 to 42% and 27 to 45% of patients, respectively. Distant metastases is most commonly present in the liver and lungs. Radiation therapy is initiated for palliative situation for non-resectable recurrences [76,77]. Ifosfamide- or doxorubicin-based regimens are suggested for recurrent adenosarcoma [76,77,87]. In conclusion, the management of UAS should be based on clinical experience, patient comorbidities and patient preference [87].

## 4. Conclusions

Uterine sarcomas raise many controversies in oncogynecological practice. Effective preoperative diagnostic modalities for these sarcomas have not been estimated yet. Molecular studies are more often used as an indispensable part of uterine sarcoma diagnosis and prognosis. Therefore, a development of molecular classification of uterine sarcomas should be consider in the future. There are sufficient data about best optimal treatment regimes, as the role of AHT and ART is controversial. It is supposed that target therapies could play a potential role in uterine sarcomas treatment, but the effects of such therapies have not yet been fully elucidated. Multidisciplinary team management is mandatory in order to increase OS, PFS and DFS of patients with uterine sarcomas. 

## Figures and Tables

**Table 1:** Prognostic factors of ULMS.

Prognostic Factors	Favorable	Unfavorable
*Initial tumor stage*	Early	Advanced
*Patient’s age*	<50 years	>50 years
*Mitotic count*	<10 mitoses	>10 mitoses
*Tumor size*	<10 cm	>10 cm
*Tumor margins*	Negative	Positive
*Race*	White	Afro-American
*Vascular space involvement*	No	Yes
*Oestrogen/Progesterone receptors*	Yes	No
*Ki-67 expression*	Low	High
*P-53 expression*	No	Yes
*P-16 overexpression*	No	Yes
*Morcellation*	No	Yes
*Cervical invasion*	No	Yes
*Locoregional metastasis*	No	Yes
*Distant metastasis*	No	Yes
*Bcl-2 expression*	Yes	No
*Percentage of necrosis*	Low	High

**Table 2:** FIGO Staging of uterine ULMS and endometrial stromal sarcoma [5,6,7,8,9,26].

**Stage I**	Tumor confined to the uterus
**IA**	Less than 5 cm
**IB**	More than 5 cm
**Stage II**	Tumor extends beyond the uterus, within the pelvis
**IIA**	Adnexal involvement
**IIB**	Involvement of other pelvic tissues
**Stage III**	Tumor infiltrates abdominal tissues (lesions must not just protrude into abdominal cavity)
**IIIA**	Tumor infiltrates abdominal tissues in 1 site
**IIIB**	Tumor infiltrates abdominal tissues in > 1 site
**IIIC**	Pelvic and/or para-aortic lymph nodes involvement
**Stage IV**	
**IVA**	Tumor invades the bladder and/or the rectum
**IVB**	Distant metastasis

**Table 3:** Prognostic factors of UAS.

Prognostic Factors	Favorable	Unfavorable
*Patient’s age*	<53	>53
*Initial tumor stage*	Early	Advanced
*Sarcomatous overgrowth*	No	Yes
*Myometrial invasion*	No, or <50%	>50%
*Morcellation*	No	Yes
*Lymph node involvement*	No	Yes
*Lymphovascular invasion*	No	Yes
*Tumor necrosis* *	No	Yes
*Cellular atypia* *	Mild	Severe

**Table 4:** FIGO staging of UAS.

**Stage I**	Tumor confined to the uterus
**IA**	Tumor confined to endometrium/endocervix with no myometrial invasion
**IB**	<50% myometrial invasion
**IC**	>50% myometrial invasion
**Stage II**	Tumor extends beyond the uterus, within the pelvis
**IIA**	Adnexal involvement
**IIB**	Other pelvic structures involvement
**Stage III**	Tumor infiltrates abdominal tissues (lesions must not just protrude into abdominal cavity)
**IIIA**	Tumor infiltrates abdominal tissues in one site
**IIIB**	Tumor infiltrates abdominal tissues in > one site
**IIIC**	Pelvic and/or para-aortic lymph nodes involvement
**Stage IV**	
**IVA**	Tumor invades bladder and/or rectum
**IVB**	Distant metastasis

## Data Availability

The authors declare that all related data are available concerning researchers by the corresponding author’s email.
